# A Guided Internet-Based Problem-Solving Intervention Delivered Through Smartphones for Secondary School Pupils During the COVID-19 Pandemic in India: Protocol for a Pilot Randomized Controlled Trial

**DOI:** 10.2196/30339

**Published:** 2021-10-06

**Authors:** Pattie P Gonsalves, Rhea Sharma, Eleanor Hodgson, Bhargav Bhat, Abhijeet Jambhale, Helen A Weiss, Christopher G Fairburn, Kate Cavanagh, Pim Cuijpers, Daniel Michelson, Vikram Patel

**Affiliations:** 1 Sangath New Delhi India; 2 School of Psychology University of Sussex Brighton United Kingdom; 3 Medical Research Council Tropical Epidemiology Group, Faculty of Epidemiology and Population Health London School of Hygiene & Tropical Medicine London United Kingdom; 4 Department of Psychiatry University of Oxford Oxford United Kingdom; 5 Department of Clinical Psychology Vrije Universiteit Amsterdam Netherlands; 6 Department of Global Health and Social Medicine Harvard Medical School Boston, MA United States

**Keywords:** randomized controlled trial, internet-based intervention, smartphone, adolescent, schools, mental health, COVID-19, app, protocol, problem-solving, intervention, teenager, young adult, India, feasibility, effective

## Abstract

**Background:**

“POD Adventures” is a gamified mental health intervention delivered via a smartphone app and supported by counsellors for a target population of secondary school students in India. This paper describes the protocol for a pilot randomized controlled trial of a remotely delivered version of the intervention in the context of COVID-19 restrictions.

**Objective:**

Our objectives are to assess the feasibility of research procedures and intervention delivery and to generate preliminary estimates of the effectiveness of the intervention to inform the sample size calculation of a full-scale trial.

**Methods:**

We will conduct a parallel, 2-arm, individually randomized pilot controlled trial in 11 secondary schools in Goa, India. This pilot trial aims to recruit 70 participants with a felt need for psychological support. Participants will receive either the POD Adventures intervention delivered over 4 weeks or usual care comprising information about local mental health services and national helplines. Outcomes will be assessed at two timepoints: baseline and 6 weeks post randomization.

**Results:**

The first participant was enrolled on January 28, 2021, and 6-week assessment completed on April 4, 2021. Owing to a second wave of the COVID-19 pandemic in India, schools in Goa were closed on April 22, 2021. Trial participants are currently receiving the intervention or completing follow-up assessments.

**Conclusions:**

This pilot trial will help understand the feasibility of implementing and evaluating a remotely delivered digital mental health intervention in a low-resource setting. Our findings will be used to design future trials that can address difficulties of accessing psychosocial support in-person and support wider efforts to scale up evidence-based mental health interventions for young people.

**Trial Registration:**

ClinicalTrials.gov NCT04672486; https://clinicaltrials.gov/ct2/show/NCT04672486

**International Registered Report Identifier (IRRID):**

DERR1-10.2196/30339

## Introduction

Globally, 10%-20% of adolescents experience mental health conditions, but the majority of them do not seek help or receive care [1,2]. The COVID-19 pandemic has increased the incidence of some mental disorders among the youth and has exacerbated existing mental health problems [3-7], with worsening mental health outcomes linked to social isolation, disrupted education, and worries about the future [8].

The pandemic has also led to rapid and large-scale changes in service provision, particularly in the transition to internet-based delivery of care [9,10]. Simultaneously, reviews of digital mental health interventions consistently raise concerns about the accessibility of digital technologies among disadvantaged groups [11] and difficulties keeping users engaged even among groups with access to technology [12]. Though promising gamified approaches have recently emerged [12,13], evidence from low-resource settings is especially scare [14,15].

The current protocol describes a pilot feasibility trial of “POD Adventures”—a novel gamified intervention delivered via a smartphone app and supported remotely by counsellors for a target population of secondary school students in India. Although the intervention was developed prior to the COVID-19 pandemic, the timing of the COVID-19 outbreak meant that the trial was launched in the midst of lockdowns and extended school closures. This required a pragmatic trial design that examined feasibility parameters related to the remote delivery and evaluation of POD adventures specifically, as well as offering insights into more general issues related to optimizing recruitment and sustaining engagement in internet-based trials and interventions.

POD Adventures is part of the PRIDE research program (2016-2022), which was conceived to address the scarcity of evidence-based interventions for common adolescent mental health problems in India and low-resource settings more broadly. This has involved the development and evaluation of a suite of transdiagnostic psychological interventions that can be delivered by nonspecialist (“lay”) counsellors in under-resourced school settings [16-18]. POD Adventures was conceptualized as an open-access, early intervention to promote adaptive coping and mitigate risks for developing more severe and socially disabling mental health problems in the longer term. The app was collaboratively designed with adolescents by using a person-centered approach [19]. The intervention integrates brief guidance from a lay counsellor with self-guided digital content from an app, in line with findings that human facilitation can enhance engagement with and outcomes of digital mental health interventions [12,20]. Co-design workshops with young people and iterative piloting suggested that the optimal delivery mode for POD Adventures involved small group sessions with up to 6 students working independently on smartphones under the supervision of a counsellor. This offline, school-based format was evaluated in 2019-2020 as part of an uncontrolled cohort study (N=248), with findings suggesting that the intervention was acceptable, engaging, and feasible to deliver in school settings [18].

This paper describes the protocol for a pilot randomized controlled trial on POD Adventures delivered in an alternative internet-based format, necessitated by COVID-19–related school closures in 2020-2021. School disruptions led us to reposition the intervention on the internet and remotely delivered for students to use at home. The intervention maintains all elements of the pre-existing digital specification, although modifications have been made for internet-based recruitment and remotely delivered guidance from counsellors (Multimedia Appendix 1). The specific objectives of this trial are to assess the feasibility of research procedures and intervention delivery and generate preliminary estimates of the effectiveness of the intervention to inform the sample size calculation for a full-scale trial.

## Methods

### Design

This protocol adheres to the Standard Protocol Items: Recommendations for Interventional Trials (SPIRIT) 2013 guidelines [21]. The study uses a parallel, 2-arm, individually randomized pilot controlled trial design. Outcomes will be assessed at two timepoints: baseline and 6 weeks post randomization.

### Setting

The trial will be conducted in partnership with 11 coeducational, government-aided, English-medium secondary schools in Goa, India, with an overall sampling frame of approximately 2500 students. Schools are relatively small with an average of 230 students within grades 9-12, which will be targeted in this study. Goa is one of India’s most urbanized states and offers a relevant context in which to evaluate a technology-enabled intervention intended for low-resource settings. The schools comprise adolescents from both centrally located urban and remote rural areas of the state.

### Eligibility Criteria

Eligible participants will (1) be enrolled in grades 9-12 (ages 13-19 years) in collaborating schools, (2) have access to an internet-enabled Android smartphone with a valid phone number for the duration of the pilot trial, (3) be able to read and understand English, and (4) provide their assent and parental consent (for participants aged <18 years).

We will exclude students who (1) are unable to understand intervention material (eg, owing to a reading or hearing disability or inability to comprehend English) and (2) are identified as having an elevated risk of self-harm or suicide and requiring external referral, based on a brief screening questionnaire and follow-up structured interview.

### Interventions

#### Intervention Arm

##### Content

POD Adventures is grounded in the stress-coping theory [22], with a mechanistic focus on problem-solving. The content of the POD Adventures app comprises two sections: “Adventures,” which teaches problem-solving concepts and methods through contextually appropriate games; and “My POD,” which scaffolds the student through the application of step-by-step problem-solving procedures to their own prioritized problems. This is built around the acronym “POD,” which corresponds to three problem-solving steps: (1) identify 1 or more current distressing or impairing problems (“Problem identification”), (2) identify ways of modifying the chosen problem or the accompanying emotional response and select the most promising option (“Option generation”), and (3) implement the chosen solution and evaluate the outcome (“Do it”) (Table 1). These problem-solving steps were originally refined and evaluated for use in nondigital intervention formats through earlier PRIDE studies [16]. The app will be provided in English with Konkani or Hindi (local language) voice-over options.

**Table 1:** Intervention overview.

Content sections	Description	Delivery
Problem identification	Problem identification and prioritization. This section includes practicing an emotion regulation exercise of “colour breathing,” a guided breathing exercise with visualization.	Individual telephone onboarding to orient the student to the app and build rapport. Independent gameplay of the app with support and troubleshooting as required.
Option generation	Generating options to solve the identified problems, learning to weigh pros and cons, and selecting the best option. This section includes practicing mindful stretching.	Independent gameplay of the app with support and troubleshooting as required.
“Do it” plan	Making a “do it” plan for selected option(s); practicing an emotion regulation exercise of “happy place”—guided imagery exercise of imagining a place the participant feels happy, safe, and calm.	Independent gameplay of the app with support and troubleshooting as required.
Review	Reviewing the outcomes of the “do it” plan and making a revised plan where necessary; practicing any emotion regulation exercise of the participant’s choice.	Individual telephone review of student’s progress and understanding of POD steps. Independent gameplay of the app with support and troubleshooting as required.

##### Delivery

The intervention is delivered individually through a combination of 1:1 telephone guidance and app use at the participants’ own time. In the first instance, participants will be directed to a dedicated study website to watch a 2-minute video that provides an overview of the app and how to use it. They will then attend a 1:1 brief telephone “on-boarding” session with a counsellor in which the counsellor offers an overview of the intervention and explores the participant’s prioritized problems. The counsellor will also provide the participant with a 4-digit app download password to download the app from the study website onto their own/shared family device. The app will be offered to participants for use at their own time over 4 weeks for a suggested minimum duration of 30 minutes per week. The app guides participants through the Adventures and My POD sections, and participants can choose to work on 1 or more self-nominated problems. They are encouraged to work at their own pace through all of the Adventures content, and with respect to at least 1 prioritized problem in My POD, over 4 weeks.

For the duration of the study period, participants will receive a weekly reminder SMS text message containing words of encouragement to use the app. They will also receive a notification to use the app if they do not log in for 5 consecutive days. On-demand telephone support from a counsellor will be available for addressing technical problems and clarifying app content throughout the study. A troubleshooting guide on app installation, resetting passwords, internet problems, and contacting the study team will be available for participants to access on the study website.

Each participant’s progress through the app will be visible to their allocated counsellor via a secure web portal. During the fourth week of the intervention or on completing the app contents, whichever is first, a brief “review” call will be arranged between the counsellor and participant via text message or a phone call to discuss the participant’s progress, overall learning, and their plan for managing future problems. Participants who require additional help after completing the intervention will be provided with a self-referral sheet containing information about local and national mental health services.

##### Counsellors

Guidance will be provided by 2 bilingual English- and Konkani-speaking lay counsellors. They have 2 years of experience in delivering a face-to-face (analogue) problem-solving intervention [17] and 1 year of experience in facilitating use of the POD Adventures app in school-based group sessions [18]. Although college graduates, the counsellors do not possess formal training in psychotherapy or experience beyond the scope of low-intensity problem-solving. The counsellors have received initial 4-day office-based training built around a structured intervention manual.

Counsellors will offer individual guidance to each participant and will comprise the scheduled on-boarding and review calls. In addition, counsellors will proactively make telephone calls to participants who do not use the app despite reminders.

Supervision will consist of weekly peer group supervision meetings (lasting approximately 1 hour), moderated by a psychologist. In each meeting, the counsellors will discuss progress of individual participants, review fidelity checklists of on-boarding and review sessions, and identify areas where troubleshooting or support might be required by any participants.

#### Control Arm

Through the study website, participants will be sent a digital flyer consisting of information and contact details about local mental health service providers and 2 recently established government provided/affiliated helplines [23,24].

### Measures

#### Participant Characteristics

At baseline, we will collect descriptive sociodemographic data about the selected school populations and adolescents registering for the study. Students will provide their name, phone number, gender (male or female), date of birth, email address (optional), grade, home address, parent/guardian contact information, school name, and how they learned about the study. Enrolled participants will also be asked to respond to 4 questions about their mobile phone and internet use relating to ownership and frequency of use.

#### Feasibility Outcomes

Feasibility of research procedures will be assessed through routinely logged numbers and proportions of eligible/ineligible self-referrals (with reasons for ineligibility), assenting/consenting participants (with reasons for not assenting/consenting), randomized participants (with reasons for not randomizing), and completed outcome assessments (with reasons for noncompletion).

Feasibility of the intervention delivery will be assessed using data on attendance, intervention completion (ie, attendance at on-boarding and review telephone calls and use of the POD Adventures app) and counsellor-completed fidelity checklists of on-boarding and review discussions.

Intervention processes will be assessed through the number and duration of contacts with counsellors, number of days between on-boarding and review sessions, amount of app content completion, and reasons for non-completion. Data about participants’ use of the app will also be captured securely from integrated analytics software. Key indicators will include login and logout timestamps, knowledge of problem-solving assessed through multiple-choice quizzes, and self-reported use of problem solving in real-world situations.

User satisfaction data will be obtained from participants in the intervention arm at 6 weeks using an 8-item service satisfaction questionnaire [25] with 4 appended forced-choice items that ask specifically about the experience of using the POD Adventures app.

After the follow-up assessment, semistructured qualitative interviews will be conducted with approximately 10-15 participants sampled purposively in accordance with sex and age from both study arms; the exact number of interview participants will depend on thematic saturation. Interviews will be carried out over the telephone by a researcher who has not been involved in intervention delivery. Participants will be asked about their experiences of internet-based research procedures such as recruitment, use of the study website, consent, and assessment procedures. Intervention arm participants will be asked additional questions about acceptability of using the intervention on the internet, their experiences of guidance from counsellors, usability and utility of app features, and potential harms. Interviews will be audio-recorded and transcribed by a member of the study team.

#### Clinical Outcomes

Clinical outcomes will be assessed using 2 validated self-report questionnaires that measure psychosocial problem severity (Youth Top Problems [YTP]) [26] and self-reported depression and anxiety (Revised Child Anxiety and Depression Scale–Short Version [RCADS-25]) [27]. Assessments will be carried out at two timepoints: prerandomization at baseline and postintervention follow up (6 weeks after randomization). Measures will be collected on the internet through the study website.

### Sample Size

We used a confidence interval approach for the calculation of sample sizes for external pilot randomized controlled trials [28] which recommend a sample size of at least 70 participants (35 per arm) to estimate the standard deviation for a continuous outcome with good precision for a pilot RCT.

### Recruitment and Consent Procedures

**Figure 1:**
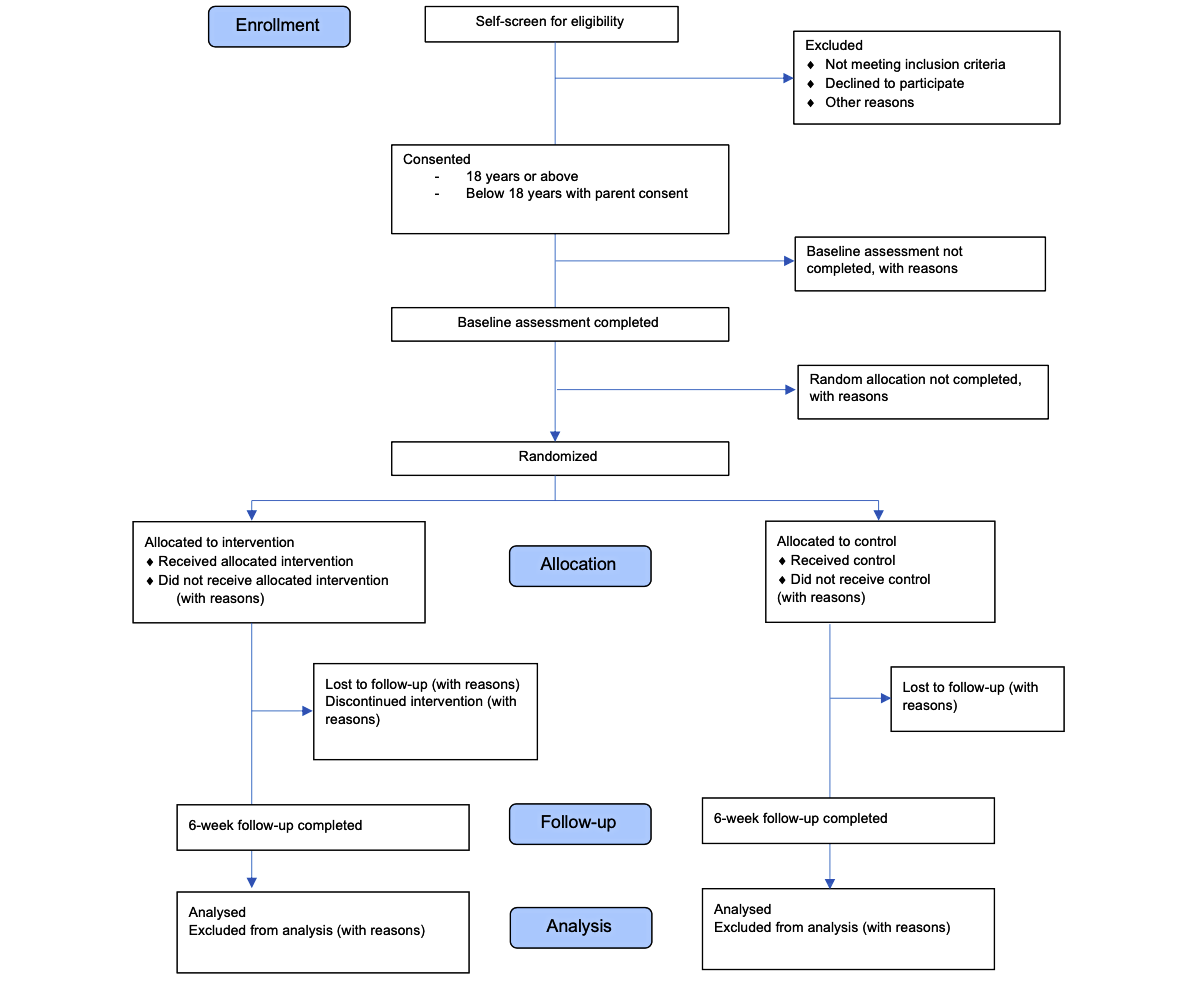
CONSORT (Consolidated Standards of Reporting Trials) flow diagram that will be used to illustrate participation throughout the phases of the POD Adventures pilot trial.

The participant flow diagram is shown in Figure 1. The sampling frame consists of all students from relevant classes in the participating schools. Recruitment will be initiated using (1) a brief 20-30–minute sensitization session delivered to individual classes either on the internet (via virtual classrooms) or, where social distancing policies allow, in school using a slideshow and brief video containing information about the study; and (2) distribution of an electronic or printed information flyer via school-moderated email/WhatsApp groups explaining the study and how to participate.

Interested students will be invited to visit the study website [29] where they will first be required to complete an eligibility assessment in accordance with the study inclusion criteria. If the student is eligible, he/she will be able to watch an animated video about the study and read information about what study participation will entail. Ineligible students will be provided with a digital information flyer that includes details about local and national services and helplines. This will be provided in a language of their choice (English, Hindi, or Konkani).

As part of the study registration process, eligible participants will be asked to provide basic demographic details and create a password for their use of the study website. Following registration, we will obtain digital consent from participants aged >18 years and assent from those aged <18 years. Parent /guardian (“caregiver”) consent will also be obtained for participants aged <18 years. Prospective participants and caregivers (if the index adolescent is aged <18 years) will be presented with information in writing, supported by an audio soundtrack in a preferred language, on the study website. The information will be followed by a series of “yes” and “no” questions to establish understanding and willingness to enroll in the study, and verified with a digital signature. For assenting participants below 18 years of age, digital parental consent will be followed by a confirmatory telephone call to the parent/guardian from the study team within 2 working days.

A toll-free helpline will also be made available for prospective participants to ask specific questions and seek technical support for registration.

### Allocation and Randomization

Each participant will be allocated a unique, anonymized ID number after registering on the study website. Upon completion of consent, a notification will be sent to the study data manager via a secure web portal designed for the study data collection. Randomization will be performed by the data manager on this platform, and the outcome of allocation will be communicated to the participants through a telephone call from a researcher and through an SMS text message alert, both of which will inform the participant to log in to the study website for information about their allocation. The study website will create a personalized dashboard that directs the participant to their next step.

The randomization algorithm will be computer-generated and stratified by school grade using randomly sized blocks of 4, 6, and 8. Participants and counsellors will not be blinded to the allocation assignment. However, other members of the research team (the principal investigator, trial statistician, and researchers) will remain blind to participation allocation status.

### Data Collection

#### Screening and Initial Assessments

The schedule for enrollment, interventions, and assessments is summarized in Table 2. Participants will complete a self-screen for eligibility and then register on the study website. They will receive an automated SMS text message alert to complete the baseline assessment once assent/consent is received. A researcher will make contact via telephone to remind the participant if the baseline assessment has not been completed 2 days thereafter. The measures take approximately 15-20 minutes to complete on the internet. Researchers will make up to 4 telephone attempts over the subsequent 2 weeks.

**Table 2:** Schedule for enrollment, interventions, and assessments.

Timepoint					Enrollment (7 working days)			Allocation (0 weeks)			Follow-up at 6 weeks post randomization
**Enrollment**											
	Self-screener for eligibility			✓							
	Informed assent (participant) and consent (parent/guardian)			✓							
	Allocation										
**Interventions**											
	POD Adventures						✓				
	Usual care information						✓				
**Assessments**											
	Demographic information			✓							
	**Outcome assessments (self-reported)**			✓						✓	
		Youth Top Problems (YTP)									
		RCADS-25									
	**Process evaluation**										
		Service satisfaction questionnaire							✓		
		App process data (intervention arm)									
		Qualitative interviews	✓			✓			✓		
		Research and intervention process data									

#### Follow-up Assessment

Participants will receive an SMS text message reminder 42 days (ie, 6 weeks) post randomization to complete the follow-up assessment on the study website. This will be accompanied by a telephone call from the researcher using a standardized script that asks participants to complete the assessment. Automated SMS text message reminders will be sent to the participants every 3 days over the next 2 weeks or until the follow-up assessment is completed on the study website. Researchers will make up to 4 telephone attempts following this due date, with a maximum allowance of 2 weeks.

#### Qualitative Interviews

Within 2 weeks of completing the follow-up assessment, a subsample of participants, purposively selected from both trial arms, will be invited via telephone to take part in an interview.

#### Strategies for Promoting Participant Compliance, Retention, and Completing Follow-up

Intervention participants' attendance at scheduled telephone sessions will be logged by counsellors. We will also undertake the following activities to support adherence to study procedures in both trial arms:

1. All participants will receive an SMS text message instruction to complete their baseline and follow-up assessments, along with SMS text message notification once this is completed.
2. All participants will receive a telephone call from a researcher 2 days after the first SMS text message alert with an invitation to complete the baseline assessment and as soon as their follow-up assessment is due.
3. If a participant cannot be reached by telephone after 4 consecutive attempts, they will be sent an SMS text message and asked to opt in for any further contact.
4. Participants in the intervention arm will receive an SMS text message reminder 1 day prior to on-boarding and review telephone sessions.
5. All telephone calls and SMS text messages, successful and unsuccessful, will be documented.

### Data Security and Management

The study will be hosted on the servers of Sangath, the implementing organization based in Goa, India. These servers will be encrypted, with data backups occurring daily. The study web portal and its associated data will be accessible only to authorized and approved personnel. When registering, participants will create password-protected accounts and the platform allocates a unique trial IDs to participants. For analyses, data will be deidentified by removing names, contact information, and any other personal identifiers. Students who withdraw from the study will have their data deleted and a withdrawal confirmation notification will be sent from the research team by telephone or email. All data will be stored securely for 10 years.

### Monitoring and Safety

#### Data Monitoring

Monitoring and governance for the pilot trial will be provided by a Trial Steering Committee (TSC; comprising senior investigators and independent subject experts) and Data and Safety Monitoring Committee (DSMC; a fully independent group with relevant clinical and trials expertise). Any study protocol amendments will be agreed and formulated in conjunction with the TSC and DSMC and submitted to relevant institutional review boards for approval.

#### Harms

The study team will continuously monitor for any participant safeguarding concerns. At baseline, all participants will be screened for risk of self-harm or suicide. Risk will be identified using a brief screening questionnaire followed by a telephone-based structured assessment where indicated. If a participant reports the presence of any thoughts of self-harm or suicide during the baseline assessment or during on-boarding or review phone calls (intervention arm participants), a risk management session will be provided to the participant within 24 hours along with information about support services will be immediately provided by the counsellor. If deemed appropriate by the clinical supervisor, the participant will also be referred to an independent mental health specialist for further assessment/treatment. At the 6-week follow-up assessment, all participants will be asked about any negative effects of using the intervention or participation in the study more generally.

#### COVID-19 Precautions

The research team will implement the pilot trial in line with local and national public health guidance and make every effort to minimize in-person visits to schools unless specifically requested by schools. Health and safety measures outlined in local government guidelines for school reopening will be strictly followed by research staff who may visit schools as part of any recruitment activities. In addition, fieldwork safety training will be provided to all study team members. Study team members will employ measures to maintain physical distancing and use of personal protective equipment such as masks in line with local health and safety protocols.

### Analyses

#### Statistical Analysis

The statistical analysis for this pilot trial will be mainly descriptive in nature, aiming to provide estimates of key trial parameters and to inform power calculations for a future trial. The outcome measures will be summarized at baseline and at 6-week follow up by trial arm. These will be summarized by mean (SD), median (IQR), or n (%) values as appropriate to relevant subgroups (defined by age, gender, and baseline outcome score). For continuous outcomes, histograms will also be plotted within each arm to assess normality and whether any transformation is required.

Analyses will be conducted to examine the effect of the intervention in normal and clinical subgroups (as measured by the RCADS-25).

#### Qualitative Analysis

Qualitative interviews will be transcribed verbatim and downloaded to NVivo (version 12, QSR International). Thematic coding frameworks will be constructed to allocate codes to emergent themes within the data, facilitating their identification and organization. Transcripts will be independently coded to enable discrepancies to be identified and consensus reached about the interpretation and application of the coding framework. Data that do not fit the initial coding framework will lead to the generation of new themes and framework revision. Data will then be consistently classified, indexed, and subject to thematic analysis using the refined coding framework.

### Ethics and Safety

Institutional review board approvals have been obtained from the Indian Council of Medical Research (ICMR); Sangath (the implementing organization in India); Harvard Medical School, Boston (Massachusetts, United States; the sponsor), London School of Hygiene and Tropical Medicine, United Kingdom (collaborator); and the University of Sussex, United Kingdom (collaborator). Permissions from individual institutions have also been obtained for all participating schools.

The principal investigator will act as the custodian of the data in accordance with the legislation of the research sponsor (Harvard Medical School) and funder (Wellcome Trust, United Kingdom).

### Dissemination Plan

School reports, consisting of the mean aggregate scores for the measures, will be prepared and shared with the school at completion of the data collection period. The study results will be prepared for academic publication in open-access mode.

## Results

Student sensitization sessions began on the internet and in person on January 11, 2021. The first participant was enrolled on January 28, 2021, and his/her 6-week assessment was completed on April 4, 2021. Owing to a second wave of the COVID-19 pandemic in India, schools in Goa were closed on April 22, 2021. At the time of manuscript submission, trial participants are receiving the intervention or completing follow-up assessments, with all activities carried out remotely.

## Discussion

This paper describes the POD Adventures pilot trial, which aims to assess the feasibility of conducting a future large-scale trial of a gamified mental health intervention for secondary school students in India. Designed as an early intervention for common youth mental health problems, POD Adventures is intended to meet the growing need for mental health support among secondary school students in India [30]. All intervention and research activities have transitioned to the internet in the context of COVID-19 restrictions. The results will therefore offer specific insights into the viability of delivering and evaluating psychosocial interventions under conditions of social distancing and school closures.

An individually randomized design was chosen for this study owing to the relatively small number of available schools, which ruled out an alternative cluster-randomized design. Risks of contamination are minimized through remote internet-based delivery, which limits potential for communication between participants that might ordinarily occur in school settings. Additionally, the choice of a usual care control, consisting of information about other services and helplines, rules out the possibility of contamination due to the same counsellors interacting with both trial arms [31].

Key challenges of this study may be uptake and adherence. Despite the compulsory transition to internet-based education for students across India, there are still variations in smartphone ownership and access to internet connectivity [32]. Young people from high-income settings have reported many challenges impacting on their engagement with internet-based interventions, such as limited access and technical issues, lack of time, doubts regarding the perceived helpfulness of the program, and preferences for face-to-face help [12]. Further, delivery of self-directed digital programs for youth at home and in other relatively unmonitored settings has been associated with relatively poor adherence [33]. A recent review of studies from Latin America showed similar challenges [34]. Low mental health literacy in our demographic may be another factor that may negatively impact uptake [35]. In anticipation of these challenges, the study uses a broad range of recruitment strategies aligned with existing best practices [15], such as in-person classroom sensitization (where possible), use of explanatory videos and flyers, and use of a toll-free telephone number for queries.

Competing demands for time may be another engagement barrier and has been previously observed in PRIDE studies conducted in Indian schools [16,18]. Counsellor guidance and reminders via SMS text messaging or app notifications offered to participants in the intervention arm may positively impact retention [36]. Looking beyond the immediate context of this study, potential implementation barriers include a shortage of suitably trained, supervised, and motivated school counsellors. To address this concern, a separate component of the wider PRIDE research program will examine the effects of a digital training curriculum on competences of prospective school counsellors to deliver an evidence-based problem-solving intervention.

The strengths of this pragmatic pilot trial include the novelty of the intervention and its pivot from in-person to internet-based delivery in a low-resource setting. Outcomes will be assessed via self-report, thereby lowering the risk of bias due to unblinded outcome assessments. The study should offer useful insights about the feasibility of remotely delivered mental health interventions for adolescents in similar contexts.

## Appendix

Multimedia Appendix 1 Intervention modifications for remote online delivery.

## Abbreviations

DSMC: Data and Safety Monitoring Committee
ICMR: Indian Council of Medical Research
RCADS-25: Revised Child Anxiety and Depression Scale–Short Version
SPIRIT: Standard Protocol Items: Recommendations for Interventional Trials
TSC: Trial Steering Committee
YTP: Youth Top Problems

## References

[1] Reiner Robert C, Olsen Helen Elizabeth, Ikeda Chad Thomas, Echko Michelle M, Ballestreros Katherine E, Manguerra Helen, Martopullo Ira, Millear Anoushka, Shields Chloe, Smith Alison, Strub Bryan, Abebe Molla, Abebe Zegeye, Adhena Beyene Meressa, Adhikari Tara Ballav, Akibu Mohammed, Al-Raddadi Rajaa M, Alvis-Guzman Nelson, Antonio Carl Abelardo T, Aremu Olatunde, Asgedom Solomon Weldegebreal, Asseffa Netsanet Abera, Avila-Burgos Leticia, Barac Aleksandra, Bärnighausen Till W, Bassat Quique, Bensenor Isabela M, Bhutta Zulfiqar A, Bijani A, Bililign N, Cahuana-Hurtado L, Malta DC, Chang JC, Charlson FJ, Dharmaratne SD, Doku DT, Edessa D, El-Khatib Z, Erskine HE, Ferrari AJ, Fullman N, Gupta R, Hassen HY, Hay SI, Ilesanmi OS, Jacobsen KH, Kahsay A, Kasaeian A, Kassa TD, Kebede S, Khader YS, Khan EA, Khan MN, Khang YH, Khubchandani J, Kinfu Y, Kochhar S, Kokubo Y, Koyanagi A, Defo BK, Lal DK, Kumsa FA, Larson HJ, Leung J, Mamun AA, Mehata S, Melku M, Mendoza W, Mezgebe HB, Miller TR, Moges NA, Mohammed S, Mokdad AH, Monasta L, Neupane S, Nguyen HLT, Ningrum DNA, Nirayo YL, Nong VM, Ogbo FA, Olagunju AT, Olusanya BO, Olusanya JO, Patton GC, Pereira DM, Pourmalek F, Qorbani M, Rafay A, Rai RK, Ram U, Ranabhat CL, Renzaho AMN, Rezai MS, Ronfani L, Roth GA, Safiri S, Sartorius B, Scott JG, Shackelford KA, Sliwa K, Sreeramareddy C, Sufiyan MB, Terkawi AS, Topor-Madry R, Tran BX, Ukwaja KN, Uthman OA, Vollset SE, Weldegwergs KG, Werdecker A, Whiteford HA, Wijeratne T, Yonemoto N, Yotebieng M, Zuhlke LJ, Kyu HH, Naghavi M, Vos T, Murray CJL, Kassebaum NJ, GBD 2017 Child and Adolescent Health Collaborators (2019). Diseases, Injuries, and Risk Factors in Child and Adolescent Health, 1990 to 2017: Findings From the Global Burden of Diseases, Injuries, and Risk Factors 2017 Study. JAMA Pediatr.

[2] Adolescent mental health. World Health Organization.

[3] Jones EAK, Mitra AK, Bhuiyan AR (2021). Impact of COVID-19 on Mental Health in Adolescents: A Systematic Review. Int J Environ Res Public Health.

[4] Galea S, Merchant RM, Lurie N (2020). The Mental Health Consequences of COVID-19 and Physical Distancing: The Need for Prevention and Early Intervention. JAMA Intern Med.

[5] CDC COVID-19 Response Team (2020). Severe Outcomes Among Patients with Coronavirus Disease 2019 (COVID-19) - United States, February 12-March 16, 2020. MMWR Morb Mortal Wkly Rep.

[6] Liang L, Ren H, Cao R, Hu Y, Qin Z, Li C, Mei S (2020). The Effect of COVID-19 on Youth Mental Health. Psychiatr Q.

[7] Serlachius A, Badawy SM, Thabrew H (2020). Psychosocial Challenges and Opportunities for Youth With Chronic Health Conditions During the COVID-19 Pandemic. JMIR Pediatr Parent.

[8] Loades ME, Chatburn E, Higson-Sweeney N, Reynolds S, Shafran R, Brigden A, Linney C, McManus MN, Borwick C, Crawley E (2020). Rapid Systematic Review: The Impact of Social Isolation and Loneliness on the Mental Health of Children and Adolescents in the Context of COVID-19. J Am Acad Child Adolesc Psychiatry.

[9] Moreno C, Wykes T, Galderisi S, Nordentoft M, Crossley N, Jones N, Cannon M, Correll CU, Byrne L, Carr S, Chen EYH, Gorwood P, Johnson S, Kärkkäinen H, Krystal JH, Lee J, Lieberman J, López-Jaramillo C, Männikkö M, Phillips MR, Uchida H, Vieta E, Vita A, Arango C (2020). How mental health care should change as a consequence of the COVID-19 pandemic. Lancet Psychiatry.

[10] Torous J, Jän Myrick K, Rauseo-Ricupero N, Firth J (2020). Digital Mental Health and COVID-19: Using Technology Today to Accelerate the Curve on Access and Quality Tomorrow. JMIR Ment Health.

[11] Taylor CB, Fitzsimmons-Craft EE, Graham AK (2020). Digital technology can revolutionize mental health services delivery: The COVID-19 crisis as a catalyst for change. Int J Eat Disord.

[12] Garrido S, Millington C, Cheers D, Boydell K, Schubert E, Meade T, Nguyen QV (2019). What Works and What Doesn't Work? A Systematic Review of Digital Mental Health Interventions for Depression and Anxiety in Young People. Front Psychiatry.

[13] Hollis C, Falconer CJ, Martin JL, Whittington C, Stockton S, Glazebrook C, Davies EB (2017). Annual Research Review: Digital health interventions for children and young people with mental health problems - a systematic and meta-review. J Child Psychol Psychiatry.

[14] Lehtimaki S, Martic J, Wahl B, Foster KT, Schwalbe N (2021). Evidence on Digital Mental Health Interventions for Adolescents and Young People: Systematic Overview. JMIR Ment Health.

[15] Liverpool S, Mota CP, Sales CMD, Čuš A, Carletto S, Hancheva C, Sousa S, Cerón SC, Moreno-Peral P, Pietrabissa G, Moltrecht B, Ulberg R, Ferreira N, Edbrooke-Childs J (2020). Engaging Children and Young People in Digital Mental Health Interventions: Systematic Review of Modes of Delivery, Facilitators, and Barriers. J Med Internet Res.

[16] Michelson D, Malik K, Krishna M, Sharma R, Mathur S, Bhat B, Parikh R, Roy K, Joshi A, Sahu R, Chilhate B, Boustani M, Cuijpers P, Chorpita B, Fairburn CG, Patel V (2020). Development of a transdiagnostic, low-intensity, psychological intervention for common adolescent mental health problems in Indian secondary schools. Behav Res Ther.

[17] Michelson D, Malik K, Parikh R, Weiss HA, Doyle AM, Bhat B, Sahu R, Chilhate B, Mathur S, Krishna M, Sharma R, Sudhir P, King M, Cuijpers P, Chorpita B, Fairburn CG, Patel V (2020). Effectiveness of a brief lay counsellor-delivered, problem-solving intervention for adolescent mental health problems in urban, low-income schools in India: a randomised controlled trial. Lancet Child Adolesc Health.

[18] Gonsalves PP, Hodgson ES, Bhat B, Sharma R, Jambhale A, Michelson D, Patel V (2021). App-based guided problem-solving intervention for adolescent mental health: a pilot cohort study in Indian schools. Evid Based Ment Health.

[19] Gonsalves PP, Hodgson ES, Kumar A, Aurora T, Chandak Y, Sharma R, Michelson D, Patel V (2019). Design and Development of the "" Smartphone Game: A Blended Problem-Solving Intervention for Adolescent Mental Health in India. Front Public Health.

[20] Grist R, Croker A, Denne M, Stallard P (2019). Technology Delivered Interventions for Depression and Anxiety in Children and Adolescents: A Systematic Review and Meta-analysis. Clin Child Fam Psychol Rev.

[21] Chan A, Tetzlaff JM, Gøtzsche PC, Altman DG, Mann H, Berlin JA, Dickersin K, Hróbjartsson A, Schulz KF, Parulekar WR, Krleza-Jeric K, Laupacis A, Moher D (2013). SPIRIT 2013 explanation and elaboration: guidance for protocols of clinical trials. BMJ.

[22] Lazarus RS, Folkman S (1984). Stress, Appraisal, and Coping.

[23] Learning Life Skills. Ministry of Education, Government of India.

[24] National Institute of Mental Health and Neurosciences.

[25] Larsen DL, Attkisson CC, Hargreaves WA, Nguyen TD The Client Satisfaction Questionnaire (CSQ) Scales. APA PsycNet.

[26] Weisz JR, Chorpita BF, Frye A, Ng MY, Lau N, Bearman SK, Ugueto AM, Langer DA, Hoagwood KE, Research Network on Youth Mental Health (2011). Youth Top Problems: using idiographic, consumer-guided assessment to identify treatment needs and to track change during psychotherapy. J Consult Clin Psychol.

[27] Klaufus L, Verlinden E, van der Wal M, Kösters M, Cuijpers P, Chinapaw M (2020). Psychometric evaluation of two short versions of the Revised Child Anxiety and Depression Scale. BMC Psychiatry.

[28] Hertzog MA (2008). Considerations in determining sample size for pilot studies. Res Nurs Health.

[29] POD Adventures.

[30] Porter C, Favara M, Hittmeyer A, Scott D, Sánchez Jiménez A, Ellanki R, Woldehanna T, Duc LT, Craske MG, Stein A (2021). Impact of the COVID-19 pandemic on anxiety and depression symptoms of young people in the global south: evidence from a four-country cohort study. BMJ Open.

[31] Magill N, Knight R, McCrone P, Ismail K, Landau S (2019). A scoping review of the problems and solutions associated with contamination in trials of complex interventions in mental health. BMC Med Res Methodol.

[32] UNICEF and the International Telecommunication Union (ITU) (2020). How many children and young people have internet access at home? Estimating digital connectivity during the COVID-19 pandemic. UNICEF.

[33] Achilles MR, Anderson M, Li SH, Subotic-Kerry M, Parker B, O'Dea B (2020). Adherence to e-mental health among youth: Considerations for intervention development and research design. Digit Health.

[34] Jiménez-Molina Á, Franco P, Martínez V, Martínez P, Rojas G, Araya R (2019). Internet-Based Interventions for the Prevention and Treatment of Mental Disorders in Latin America: A Scoping Review. Front Psychiatry.

[35] Gaiha SM, Taylor Salisbury T, Koschorke M, Raman U, Petticrew M (2020). Stigma associated with mental health problems among young people in India: a systematic review of magnitude, manifestations and recommendations. BMC Psychiatry.

[36] Grist R, Porter J, Stallard P (2017). Mental Health Mobile Apps for Preadolescents and Adolescents: A Systematic Review. J Med Internet Res.

