# Supplementary material for: A Guided Internet-Based Problem-Solving Intervention Delivered Through Smartphones for Secondary School Pupils During the COVID-19 Pandemic in India: Protocol for a Pilot Randomized Controlled Trial
Source: JMIR Res Protoc. 2021 Oct 6;10(10):e30339. doi: 10.2196/30339 (PMC8496682; doi:10.2196/30339)
Supplement: Multimedia Appendix 1 [file resprot_v10i10e30339_app1.docx]

**Multimedia Appendix 1.** Intervention modifications for remote online delivery.

| **Intervention features** | **Modification for remote online delivery** |
| --- | --- |
| *Location* | |
| POD Adventures app offered on researcher-provided smartphones on school premises during designated times. | - POD Adventures app made available for remote online download on participant’s personal device |
| *Risk assessment and management* | |
| - Risk management and assessment session conducted in person - Risk detected by researcher during baseline assessment and referred to counsellor - In-app game pausing if any potential risk detected | - Risk management and assessment session conducted telephonically - Risk detected by data manager and referred to counsellor - In-app game data not screened for risk and in-app game pausing removed |
| *Study enrolment* | |
| - Sensitisation sessions conducted for school staff and students in-person - Self-referral slips provided to students to complete and submit to researcher or place in dropboxes on school premises or walk-in to counsellor’s room - The process of getting and following up with a referral were all done in person via the researcher in the school | - Sensitisation sessions conducted for school staff and students, either in-person and remotely via virtual classroom - For in-person student sensitization, an introductory flyer and referral slip are given to each student in person - For virtual sessions, the flyer is emailed/sent via WhatsApp to all students - The process of registration is via our website-POD Adventures |
| *Intervention materials* | |
| - POD Adventures app provided on researcher device | - No device provided; intervention to be used by participant on self-owned device - Inclusion of brief onboarding video and review video using scripts used by counsellor for in-person school delivery |
| *Counsellor guidance* | |
| Intervention on-boarding (at intervention start) and review (at completion of the intervention) conducted either individually or in small groups | Intervention on-boarding and review sessions conducted individually via telephone |
| On-hand troubleshooting help for technical or content issues available during intervention sessions in school | - Troubleshooting guides available on the POD website for troubleshooting the app, troubleshooting the POD website and for game installation - Toll free number and counsellor number for any other help needed- telephonic help |
| No usage reminders to students as intervention provided in school and on researcher device | Notifications and reminders for download, weekly usage reminder and reminder for no log in for 5 days programmed |
| No tailored in-app encouragement offered | Automated weekly push notifications will offer in-app encouragement and motivation |

| ***Intervention feature*** | ***Description*** | ***Modification for remote online delivery*** |
| --- | --- | --- |
| ***General*** |  |  |
| POD Adventures app offered on researcher-provided smartphones in school |  | POD Adventures app made available for remote online download on participant’s personal device |
| Risk management and assessment session conducted in person |  | Risk management and assessment session conducted telephonically |
| ***Location*** |  |  |
| Intervention provided to participants for use in school in pre-scheduled sessions |  |  |
| ***Guidance*** |  |  |
| Intervention On-boarding session conducted in small ‘lab’ style group of up to 6 participants |  | Intervention On-boarding session conducted telephonically for each individual participant.  Brief on-boarding video designed for intervention participants. |
| No usage reminders |  |  |
| No tailored in-app encouragement offered |  |  |
|  |  |  |
